# Hyperglycemia aggravates ischemic brain damage *via* ERK1/2 activated cell autophagy and mitochondrial fission

**DOI:** 10.3389/fendo.2022.928591

**Published:** 2022-08-05

**Authors:** Ping Liu, Xiao Yang, Jianguo Niu, Changchun Hei

**Affiliations:** ^1^ Department of Endocrinology, General Hospital of Ningxia Medical University, Yinchuan, China; ^2^ Neuroscience Center, General Hospital of Ningxia Medical University, Yinchuan, China; ^3^ Ningxia Key Laboratory of Cerebrocranial Disease, Ningxia Medical University, Yinchuan, China; ^4^ Department of Human Anatomy, Histology and Embryology, Ningxia Medical University, Yinchuan, China

**Keywords:** hyperglycemia, cerebral ischemic injury, ERK1/2, Drp-1, mitochondrial fission, cell autophagy

## Abstract

**Background:**

Hyperglycemia is one of the major risk factors for stroke and stroke recurrence, leading to aggravated neuronal damage after cerebral ischemia/reperfusion (I/R). ERK1/2 signaling pathway plays a vital role in cerebral ischemic injury. However, the role of the ERK1/2 pathway in hyperglycemia-aggravated ischemic brain damage is not clear.

**Methods:**

Streptozotocin (STZ; 50 mg/kg)-induced diabetes (blood glucose ≥12 mmol/L) or control groups in adult Sprague-Dawley rats were further subdivided into I/R (carotid artery/vein clamping), I/R + PD98059 (I/R plus ERK1/2 inhibitor), and Sham-operated groups (n = 10 each). Neurobehavioral status (Neurological behavior scores) and the volume of the cerebral infarction (TTC staining); brain mitochondrial potential (JCI ratio test) and cell apoptosis (TUNEL assay); RAS protein expression, phosphorylated/total ERK1/2 and Drp-1 (Dynamic-related protein 1) protein levels (Western blotting); mitochondrial fusion-related proteins mitofusin-1/2 (Mfn1/2), optic atrophy (OPA-1) and mitochondrial fission 1 (Fis1), and autophagy-associated proteins Beclin-1, LC3-I/II and P62 (Western blotting and immunohistochemistry) were analyzed.

**Results:**

The I/R + PD98059 group demonstrated better neurobehavior on the 1^st^ (p < 0.05) and the 3^rd^ day (p < 0.01) than the I/R group. Compared to the Sham group, cerebral ischemia/reperfusion brought about neuronal damage in the I/R group (p <0.01). However, treatment with PD98059 showed an improved situation with faster recovery of mitochondrial potential and less apoptosis of neuronal cells in the I/R + PD98059 group (p < 0.01). The I/R group had a higher-level expression of RAS and phosphorylated ERK1/2 and Drp-1 than the diabetes mellitus (DM) group (p < 0.01). The PD98059 treated group showed decreased expression of p-ERK1/2, p-Drp-1, Fis1, and Beclin-1, LC3-I/II and P62, but increased Mfn1/2 and OPA-1 than the I/R group (p < 0.01).

**Conclusion:**

Hyperglycemia worsens cerebral ischemia/reperfusion-induced neuronal damage *via* ERK1/2 activated cell autophagy and mitochondrial fission.

## Materials and methods

### Ethics statement

All the Sprague-Dawley rats (specific pathogen-free, male), aged 4~6 weeks, were provided by our hospital’s animal center. All rats were housed at an appropriate temperature (22-26°C), humidity (40-70%), adequate light, and diet according to international animal ethics. All the operations were reviewed and approved by the Animal Care and Use Committee of our hospital.

### Grouping

Sprague-Dawley rats were randomly divided into three groups:diabetic non-ischemia control group(sham),diabetic ischemia/reperfusion (I/R) group,and diabetic ischemia-reperfusion+ PD98059 treatment group.The ischemia group wa sdivided into 3 subgroups of 1 day,3 days and 7 days according to the reperfusion time.Each subgroup consisted of 10 male rats.ERK1/2 inhibitors(PD98059,ab146592,Abcam) were dissolved in normal saline and injected intraperitoneally (10 mg/kg) once a day from 3 days before surgery until they were sacrificed.Rats in the non-treated group were injected with the same amount of normal saline.

### STZ -induced diabetic rat

Streptozotocin (STZ) was dissolved in fresh nicotinamide (1 ml, 0.1 mol/L, PH = 4.5) and subcutaneously injected (50 mg/kg) to yield rats with blood glucose levels of 12 mmol/l (396 mg/dl) or above on the 3^rd^ day. Four to six weeks later, these rats were further administered for the cerebral I/R rat model.

### Middle cerebral artery occlusion

An MCAO surgical operation induced cerebral I/R at 4 to 6 weeks after STZ injection. The rats were anesthetized by intraperitoneal injection of 10% chloral hydrate (350 mg/kg) and were then fixed in a supine position. The middle part of the ventral neck was treated to expose blood vessels for surgical procedures. After incision, the right common carotid artery, external carotid artery, and internal carotid artery were separated from surrounding tissues. During the process of separation, electrical coagulation breaks small branches of blood vessels to prevent bleeding. Subsequently, the external carotid artery was ligated 1 cm from the common carotid artery’s bifurcation and seared with an electric coagulator. The right common carotid artery was clipped with a clip. A small slanted opening was cut using a vessel clipping about 0.5 cm from the external carotid artery’s proximal end, which was pulled to align with the internal carotid artery. The nylon thread was slowly pushed through the right external carotid artery’s main incision to the internal carotid artery’s cranial entry direction. The bifurcation of the common carotid artery was marked. The middle cerebral artery was blocked when the common carotid artery’s bifurcation was pushed 18 to 20 mm, or a slight resistance was felt. After a 2-hour’s occlusion, ether anesthesia was used to expose the neck incision, and the threaded plug was carefully pulled out. The external carotid artery residue was tied tightly, and the subcutaneous tissue and skin were sutured. Intraoperative incandescent lamp heating was used to maintain the anal temperature at 36.5~37.0°C. Animals were sacrificed, and brain tissue was isolated for further detections.

### Assessment of neurological behavior scores

Both histological and neurological evaluations are essential for MCAO model. The neurological deficit scores were performed at 24h, 48h and 72h h after cerebral ischemia based on the previous procedure described by Bederson et al. (1). Briefly speaking, the score was assessed as follows, 0: Neurological function is normal; 1: Mild neurological impairment (Flexion of the left forelimb during tail lifting); 2: Moderate neurological impairment (Turn to the left when walking); 3: Moderate neurological deficit (incline to the left); 4: No spontaneous walking, loss of consciousness; 5: Death related to ischemia, conduct an assessment.

### Triphenyl tetrazolium chloride staining

The celebral I/R induced infarct volume was measured 24 hours after MCAO. Brain tissue was sectionally cut into 2 mm-thick coronal slices and then TTC staining was performed according to the manufacturer’s introductions (362883, Sigma). In brief, the slices were incubated in 2% TTC at 37°C for 30 min and soaked in 4% paraformaldehyde for 24 h. Then the pictures were taken and the infarct area was analysed by ImageJ software.

### Brain mitochondrial isolation and membrane potential test

At indicated times after cerebral I/R, the rats were decapitated under anesthesia, and the brain tissues were placed into the iced buffer (1 mM PMSF). All the steps of the experiment were carried on an iced condition. The brain tissue (about 50~100 mg) was dissected out and put in a 1.5 ml Eppendorf tube for weighing. After rinsing with PBS (phosphate buffer solution), the tissue was cut into pieces and placed in the iced tenfold PBS volume for 3 minutes. After the first brief centrifugation at 600 g for 10 ~ 20 seconds (4°C), the supernatant was discarded, and the precipitate was dissolved into an eightfold volume of trypsin for 20 minutes. After the second same centrifugation, the precipitate was resuspended with a twofold mitochondrial separation reagent (2). After the third same centrifugation, the precipitate was homogenized into eightfold ice mitochondrial separation reagent 20~30 times. After the fourth centrifugation at 600 g for 5 minutes (4°C), the supernatant was further centrifugated at 11000g for 10 minutes (4°C). The supernatant was removed, leaving the mitochondria-enriched pellet in the bottom of the Eppendorf tube for further analysis. The concentration was measured by BCA assay (ab102536, Abcam).

### JC-1 mitochondrial membrane potential assay

The mitochondrial suspension was incubated with TMRM fluorescent probe for 30 minutes and measured by fluorescence spectrometer according to the manufacturer’s introductions (ab113850, Abcam). The prepared JC-1 staining solution was diluted 5 times with JC-1 Buffer (1×). The purified mitochondria with a total protein of 10-100 μg were added into the 5-fold dilution of the JC-1 staining solution. The excitation wavelength and emission wavelengths are 530 nm and 573 nm, respectively. FCCP (5 um) could depolarize the mitochondrial membrane potential and be added as positive control before the experiment. Professor Andy Li assisted this part.

### TUNEL assay

According to the manufacture’s instructions (ab66108,Abcam), the brain cells were rinsed with 1 x PBS, incubated in ice with membrane penetrating solution, and then treated with protease K. After treated with 100 ul of equilibrium solution (10 min), 50 ml TDT reaction solution (37°C,1 hour) and 2 x SSC solution were added in order. The samples were treated with encapsulating solution containing Propidium Iodide (PI) and further observed under a confocal microscope. The positive cells are green, and the nuclei red.

### Western blot

The brains of rats were sacrificed under anesthesia, and the cortex and hippocampus tissues were separated. The nucleus, cytoplasm, and mitochondria subcomponents of homogenized brain tissues were separated by gradient centrifugation. Each sample’s total protein was extracted by adding 1 mL cell lysate (containing protease inhibitors, P0013J, Beyotime Biotechnology, Shanghai, China) for 45 minutes, lysed at 4°C, spun at 8000 r/min for 30 minutes, and mixed every 10 minutes. The protein concentration was determined using a BCA kit (ab102536, Abcam). Fifty microgram protein of each sample was separated by 10% SDS-PAGE (Sodium dodecyl sulfate-polyacrylamide gel electrophoresis) and transferred to polyvinylidene difluoride (PVDF) membranes (66485, PALL, USA). Subsequently, the membranes were blocked by 5% nonfat milk at room temperature for 2 hours. After washing for three times (10 min/time) with Tris-buffered saline Tween-20 (TBS/T), the membranes were probed overnight at 4°C using antibodies including RAS (67648, CST), t-ERK1/2 (9102, CST), p-ERK1/2 (4376, CST), t-Drp-1 (8570, CST), p-Drp-1 (4494, CST), Fis1 (ab96764, Abcam), OPA-1 (67589, CST), Mfn1/2 (ab57602, Abcam), LC3 I/II (12741, CST), Beclin-1 (ab210498, Abcam) and P62 (ab109012, Abcam). Internal reference antibodies include β-actin (ab8226, Abcam) in the cytoplasm, COX-IV in mitochondria, and Histon-3 in the nucleus. As described, the membranes were rinsed, and diluted horseradish peroxidase (HRP) labeled anti-rabbit or anti-mouse IgG secondary antibody (HRP-secrondary antibody anti-rabbit, 14709s, CST; HRP-secrondary antibody anti-mouse, 14708s, CST) was added for 1-hour incubation at room temperature. The membranes were rinsed as described and immersed into ECL solution (BM101, Biomiga, USA) at room temperature for 1 min. After exposure to X-ray film in the dark, the images were taken on the Odyssey camera. The results were further obtained by band density analysis.

### Immunohistochemistry

The dewaxed brain tissues (coronal level, Bregma-3.8 mm as standard) were blocked with 0.3% H_2_O_2_ or BSA. The primary antibodies were added to the slices at 4°C overnight. The next day, the washed slices were incubated with secondary antibody at room temperature for 1 hour and then incubated with ABC reaction solution for 30min. Peroxidase substrates were added and observed under a confocal microscope. Primary antibodies included antibodies targetting p-ERK1/2 (4376, CST), p-Drp-1 (4494, CST), Fis1 (ab96764, Abcam), Mfn1/2 (ab57602, Abcam), OPA1 (67589, CST), Beclin-1 (ab210498, Abcam), LC3 I/II (12741, CST), and P62 (ab109012, Abcam).

### Mitochondrial DNA copy number assay

According to the manufacture’s instructions (ScienCell, R8938), allow vials (Cat #R8938a and #R8938b) to warm to room temperature. Centrifuge the vials at 1,500 x g for 1 minute. Add 200 µl nuclease-free H2O (Cat #8938c) to mtDNA primer set (lyophilized, Cat #R8938a) to make mtDNA primer stock solution. Then add 200 µl nuclease-free H2O (Cat #8938c) to SCR primer set (lyophilized, Cat #R8938b) to make SCR primer stock solution. For each genomic DNA sample, prepare two qPCR reactions, one with mtDNA primer stock solution, and one with SCR primer stock solution. Prepare 20 µl qPCR reactions for one well. The qPCR reaction wells were sealed after 20 µl qPCR reactions were prepared for each well. Centrifuge the plates or tubes at 1,500x g for 15 seconds. The assay was performed in triplicate for each sample.

### ATP assay

According to the manufacture’s instructions (S0026, Beyotime Biotechnology), Each sample’s total protein was extracted by adding 150 ul cell lysate, mixed at 4°C, spun at 12000 x g for 5 minutes to get the supernatant. The protein concentration was determined using the BCA assay kit (ab102536, Abcam). The ATP standard solution was diluted with the ATP detection lysate into concentration gradients of 0.01, 0.03, 0.1, 0.3, 1, 3 and 10 μM. The ATP detection working fluid was then prepared. Add 100 µl of ATP working solution to the detection hole. Lay up at room temperature for 5 minutes. Then add 20ul of sample or standard to the test hole,and the RLU values were determinded by luminometer, and normalized by the protein content.

### Statistical analysis

The data was analyzed on the SPSS21.0 version (IBM SPSS Statistics, Chicago, IL, USA) and presented as mean ± SD (standard deviation). Parameterized results in multiple groups were analyzed using bivariate (blood glucose, drug therapy) variance (ANOVA). Non-parameterized results in multiple groups were compared using the Kruskal-Wallis variance and using the Mann-Whitney test in two groups.

## Introduction

Hyperglycemia is frequent in patients with cerebrovascular disease (3). Hyperglycemia and diabetes not only increase the incidence of cerebrovascular disease but aggravate brain damage, accelerate the time of injury, cause red brain infarction and increase the incidence of epilepsy after stroke (4–6). Animal experiments confirmed that the brain damage caused by stroke was increased when the blood glucose was 12-16 mmol/L, and ischemic seizures occurred simultaneously (7, 8). All animals died of status epilepticus 24 hours after cerebral ischemia-reperfusion (I/R) when the blood glucose increased to > 20 mmol/L. In diabetic patients with cerebral ischemic stroke, the mortality and disability rate increased (9–11). At present, the mechanism of aggravating brain injury caused by diabetes has not been clarified. There is a lack of effective means to treat or prevent the aggravated cerebral ischemic injury caused by diabetes.

Mitogen active protease (MAPK) plays an important role in regulating cell growth, division, and death. The extracellular signal-regulated kinase (ERK1/2) is a member of the (MAPK) family of mitogen-activated protein kinases. ERK1/2 promotes cell division and proliferation in normal development and physiological processes. The role of ERK1/2 in cerebral ischemic injury aggravated by diabetes has not been reported. It has been reported that the inhibition of the ERK1/2 pathway reduces the infarct size caused by middle cerebral artery occlusion (12). Studies have confirmed that cytokine-like factor (CLK1) inhibitors can inhibit the expression of MAPK, including ERK1/2, and reduce the inflammatory response and the infarct size caused by cerebral ischemia (13). A study on cancer cells found that ERK1/2 can cause mitochondrial fission through dynamic associated protein (Drp-1) phosphorylation, which is a key step in causing mitochondrial matrix protein leakage, activating the cell death pathway (14).

The activated ERK1/2 pathways after hyperglycemia cerebral ischemia are usually unique. In diabetic and hyperglycemic animals, cerebral ischemia caused nearly 100% death of neurons in the hippocampal CA1 region and damage to hippocampal CA3, dentate nucleus, and cortical cingulate gyrus. However, in normoglycemic animals, cerebral ischemia only caused damage in the CA1 area (8, 15). Correspondingly, the phosphorylated ERK1/2 in the CA1 area increased in the normal model and increased in the CA3, dentate nucleus, and cortical cingulate gyrus under the condition of hyperglycemic cerebral ischemia (16–20). Therefore, ERK1/2 is highly expressed in the region with dead cells, which is further confirmed by the use of ERK1/2 inhibitor U0126 that effectively inhibited the inflammatory response induced by cerebral ischemia and reduced cell injury (20, 21). Up to now, whether inhibition of ERK1/2 can reduce the hyperglycemia aggravated cerebral ischemic injury is not clear.

Normally, mitochondria are constantly in a dynamic balance between fusion and fission, with a long reticular structure in the cell. The fusion process is regulated by mitofusin 1 fusion protein 1, 2 (Mfn1/2) and optic nerve dystrophy protein (OPA1), while the fission process is regulated by dynamic related protein 1 (Drp-1), fission protein 1 (Fis1), and Endophilin B1 (Endo B1) (22).The disorder of mitochondrial dynamic balance can lead to cellular dysfunction (23). The excess fission process leads to the cleavage of mitochondrial fragmentation and the open of mitochondrial membrane permeable pore (MPTP), which further results in cell death (24, 25). Recent studies have confirmed that Drp-1 plays its damage role by transferring pro-apoptotic protein Bax to mitochondria, causing MPTP to open and cytochrome C released from mitochondria to the cytoplasm (26). Furthermore, inhibition of Drp-1 protects neural cells from injury (27).

Studies have shown that the RAS-ERK1/2 pathway causes mitochondrial cleavage and dysfunction *via* the phosphorylation of the Drp-1 serine 616 (28, 29). The inhibition of ERK1/2 inactivates Drp-1 and promotes mitochondrial fusion (29). Our previous studies have confirmed that hyperglycemic cerebral ischemia activated ERK1/2, and the inhibition of ERK1/2 reduced the degree of brain injury in rats (30, 31). According to these studies, we speculated that mitochondria are one of the important targets of hyperglycemic cerebral ischemic injury.

## Results

### The neuroprotective function of ERK1/2 inhibitor on cerebral I/R Rats

**Figure 1:**
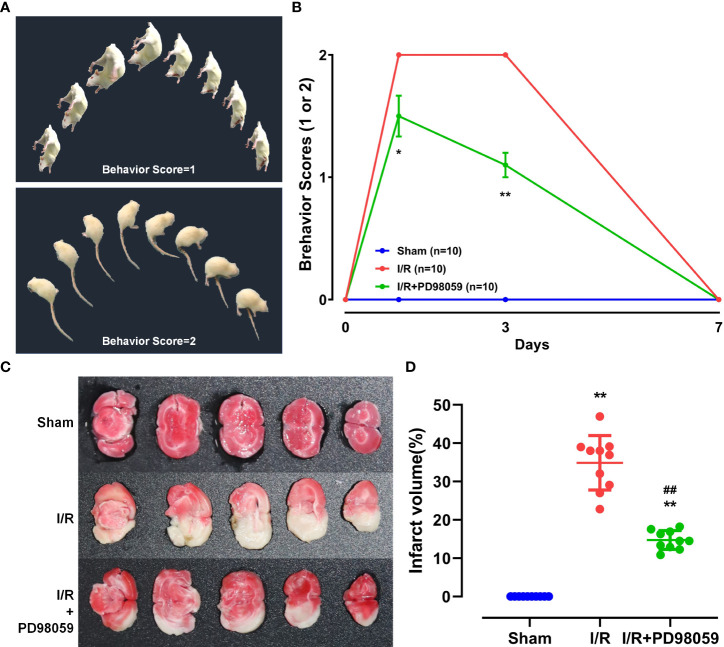
Neuroprotective function of ERK1/2 inhibitor on diabetic Rats. After cerebral I/R, rats’ behavioral ability was evaluated by behavioral score and the infarct volume by TTC staining. **(A)** The behavioral score includes the schematic diagram of score 1 and 2. **(B)** Behavioral ability on the 0^th^, 1^st^, 3^rd^, and 7^th^ day was compared between group I/R and I/R + PD98059 (Mean ± SD, n = 5). *p < 0.05; **p < 0.01. The celebral I/R induced infarct volume in different groups was determined by TTC staining **(C)** and the difference was calculated (Mean ± SD, n = 5) **(D)**. **p < 0.01, I/R + PD98059 or I/R vs Sham; ^##^p < 0.01, I/R + PD98059 vs I/R.

After cerebral ischemia-reperfusion (I/R) injury in diabetic rats, rats’ behavioral ability of I/R + PD98059 group (Upper, **Figure 1A**) and I/R (Lower, **Figure 1A**) was evaluated by neurological behavior scores according to the score of severe nerve injury. As shown in **Figure 1B**, a higher neurological function score was found in the I/R + PD98059 group compared with that of the I/R group on the 3^rd^ (p < 0.05) and 7^th^ (p < 0.01) day, respectively.

To determine celebral I/R induced infarct volume in different groups, TTC staining was performed. The normal area of brain tissue stained red, and the infarct part appeared white (**Figure 1C**). The infarct volume in group I/R and I/R + PD98059 were remarkably larger than group sham, but PD98059 treatment reduced the infarct volume from 35% to 15% (p < 0.01) (**Figure 1D**), suggesting that PD98059 improved the cerebral I/R-induced neural damage.

**Figure 2:**
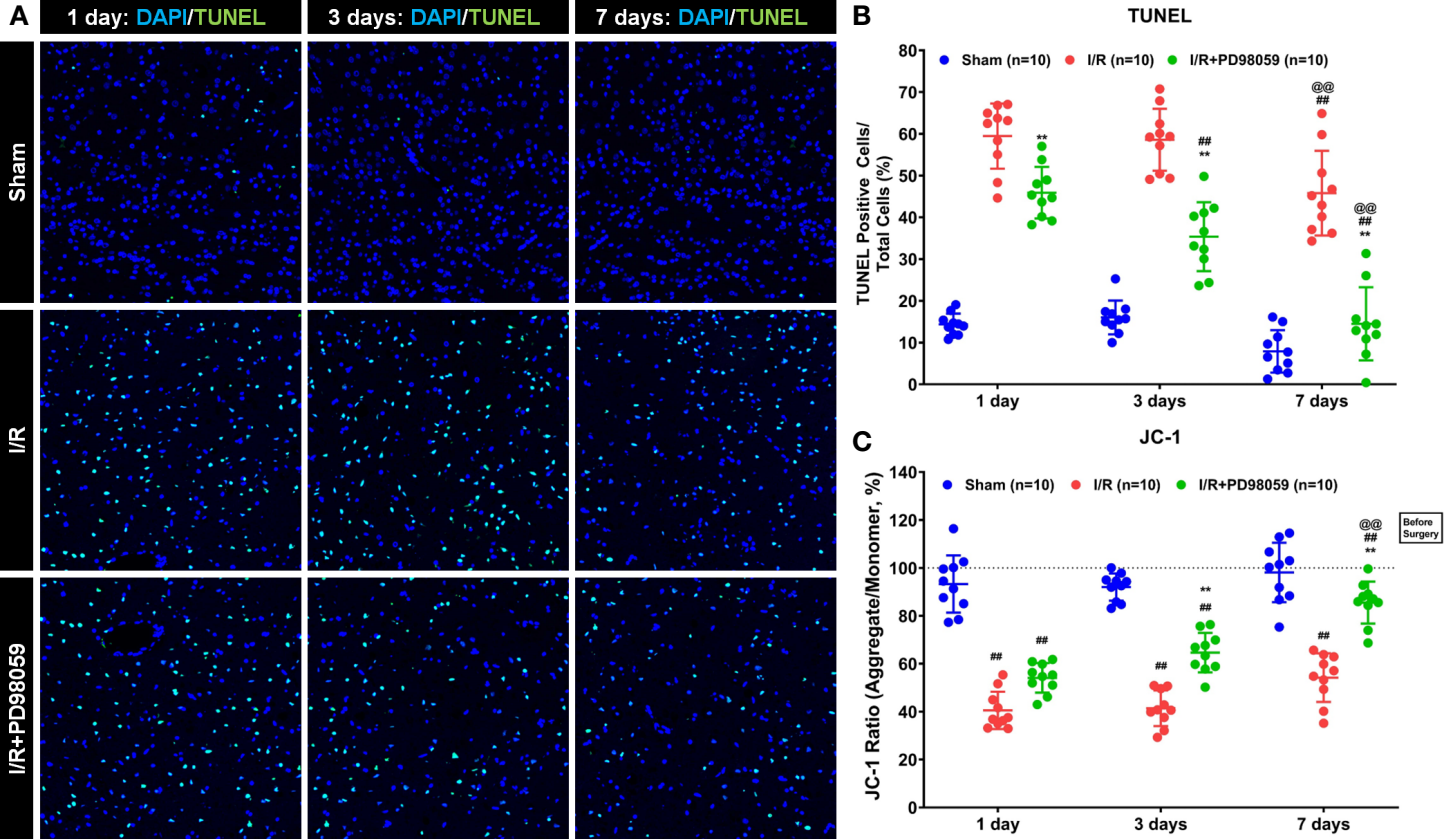
Effects of PD98059 on brain mitochondrial potential and apoptosis in diabetic rats. After I/R injury in diabetic rats, 10 diabetic rats were treated with mock or PD98059, respectively. Ten sham-operated (Sham) rats were used as the control group. The number of cell apoptosis and membrane mitochondrial potential were determined using TUNEL assay **(A, B)** and JC-1 analysis **(C)** were determined after treatment on the 1^st^, 3^rd^, and 7^th^ day. Each set of data is Mean ± SD, n = 10, compared with I/R, **p < 0.01; Compared with I/R group treated for 1 day, ^##^p < 0.01; Compared with I/R group treated for 3 days, ^@@^p < 0.01.

Previous studies have shown that hyperglycemia induces molecular signatures of mitochondrial dysfunction in neurons, including oxidative stress and apoptosis (7, 25, 26). To investigate the effects of PD98059 on mitochondrial function, the mitochondrial membrane potential and apoptosis in brain tissue of diabetic rats were analyzed and compared with the sham-operation group *via* TUNEL assay. Cerebral I/R caused a large number of apoptosis (compared with the Sham group, p < 0.05), and the number of apoptotic cells decreased from 1^st^ day to 7^th^ day (**Figures 2A, B**). However, the neural apoptosis in the group treated with PD98059 was much lower than that in the I/R group (**Figure 2B**). The above data showed that ERK1/2 inhibitor administration might play a neuroprotective function by maintaining the mitochondrial membrane potential and reducing cell apoptosis.

To further verify the effect of PD98059 on apoptosis in the brain tissue of I/R rats, the number of apoptotic cells was measured by JC-1 staining (**Figure 2C**). The results showed that compared with the Sham group, the JC-1 ratio decreased in the I/R group and the I/R + PD98059 group (**Figure 2C**), demonstrating that ischemia-reperfusion did affect the mitochondrial activity in the brain tissue of rats. The JC-1 ratio gradually recovered on the 1^st^, 3^rd^, and 7^th^ day, and the JC-1 ratio in the I/R + PD98059 group recovered faster than that in the I/R group and was similar to that in the Sham group on the 7^th^ day (**Figure 2C**). The above data showed that ERK1/2 inhibitor administration might play a neuroprotective function by maintaining the mitochondrial membrane potential and reducing cell apoptosis.

### Activation of ERK1/2 pathway after cerebral I/R in diabetic rats

**Figure 3:**
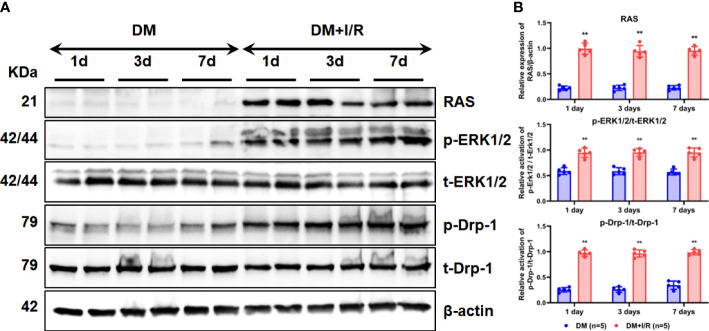
Activation of ERK1/2 pathway after cerebral I/R in diabetic rats. After I/R injury in diabetic rats, the expression and activation of RAS, ERK1/2, and Drp-1 in rat brain tissue on the 1^st^, 3^rd^, and 7^th^ day were detected by Western blot **(A)**, and gray values were determined by ImageJ **(B)**. In each group (n = 5), all the results are Mean ± SD, compared with DM group, **p < 0.01. DM, diabetes mellitus.

To test our hypothesis and explore the activation of the ERK1/2-related signaling pathway as well as the downstream regulatory molecules after cerebral I/R in diabetic rats, the expression and activation of RAS, ERK1/2, and Drp-1 were detected using western bloting. RAS expression increased in the brain tissue of diabetic rats in the diabetes mellitus (DM) + I/R group than the DM group (**Figure 3A**). Compared with the DM group, the DM + I/R group showed similar expression of t-ERK1/2 and its immediate downstream molecular t-Drp-1, but a higher expression level of RAS, p-ERK1/2 and p-Drp-1 (p < 0.01) (**Figures 3A, B**). In summary, cerebral I/R activated the ERK1/2 pathway in diabetic rats.

### ERK1/2 inhibitor modulated mitochondrial balance

With the ERK1/2 pathway activation, phosphated ERK1/2 can rapidly translocate to mitochondria and regulate a series of mitochondrial-related cellular activities (14). Mitochondria are in a dynamic balance between mitochondrial fusion and mitochondrial fission (14, 24). The mitochondrial fusion process is regulated by mitochondrial fusion protein 1, 2 (Mfn1/2) and optic nerve dystrophy protein (OPA1) (22, 29). Mitochondrial fission is regulated by dynamic related protein 1 (Drp-1) and fission protein 1 (Fis1) (22). The disorder of mitochondrial dynamic balance can lead to cell dysfunction (14, 22, 23).

**Figure 4:**
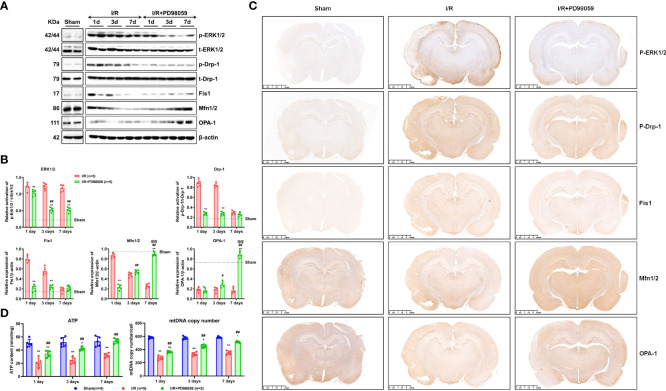
ERK1/2 inhibitor modulated mitochondrial balance. After I/R injury and administration of PD98059 in sham and diabetic rats, the phosphorylation level of ERK1/2 and Drp-1, and the expression of Fis1, Mfn1/2, and OPA1 on the 1^st^, 3^rd^, and 7^th^ day were detected using western blot **(A)**, and gray values were determined by ImageJ **(B)**. The expression of ERK1/2, Drp-1, Fis1, Mfn1/2, and OPA1 was also analyzed *via* immunohistochemistry (IHC) **(C)**. The mitochondrial DNA copy number and the ATP content were detected using Mitochondrial DNA copy number and ATP assay kits **(D)**. In each group, n = 5, all the results are Mean ± SD, compared with I/R, *p < 0.05, **p < 0.01; compared with the group of I/R + PD98059 treated for 1 day, ^#^p < 0.05, ^##^p < 0.01; compared with the group of I/R + PD98059 treated for 3 days, ^@@^p < 0.01.

To understand the protective function of ERK1/2 inhibitor on cerebral I/R Rats, the phosphorylation level of ERK1/2 and Drp-1, and the expression of ERK1/2, Drp-1, Fis1, Mfn1/2, and OPA1 on the 1^st^, 3^rd^, and 7^th^ day were detected using western blot (**Figures 4A, B**). The expression of p-ERK1/2, p-Drp-1, and Fis1 was higher, but Mfn1/2 and OPA1 were lower in the I/R group than I/R + PD98059 group (**Figure 4A**). The expression of p-ERK1/2, p-Drp-1, Fis1, Mfn1/2, and OPA1 was further confirmed *via* immunohistochemistry (IHC), showing a similar expression model between group I/R, I/R + PD98059 and sham (**Figure 4C**).

To confirm the significance of mitochondria in above process, the mitochondrial DNA copy number and ATP content on the 1^st^, 3^rd^, and 7^th^ day were evaluated. As illustrated (**Figure 4D**), mtDNA copy number and ATP content were downregulated in the I/R group compared with the sham group, but PD98059 improved the mitochondrial biomarkers in the I/R + PD98059 group, indicating mitochondria played vital role in ERK1/2-involved cell dysfunction. To sum up, ERK1/2 inhibitor PD98059 maintained mitochondrial balance by the upregulated Mfn1/2 and OPA1 and downregulated p-ERK1/2, p-Drp-1 and Fis1, suggesting that hyperglycemia worsened celebral I/R induced brain damage *via* ERK1/2 activated mitochondrial fission.

### ERK1/2 inhibitor regulated cell autophagy

**Figure 5:**
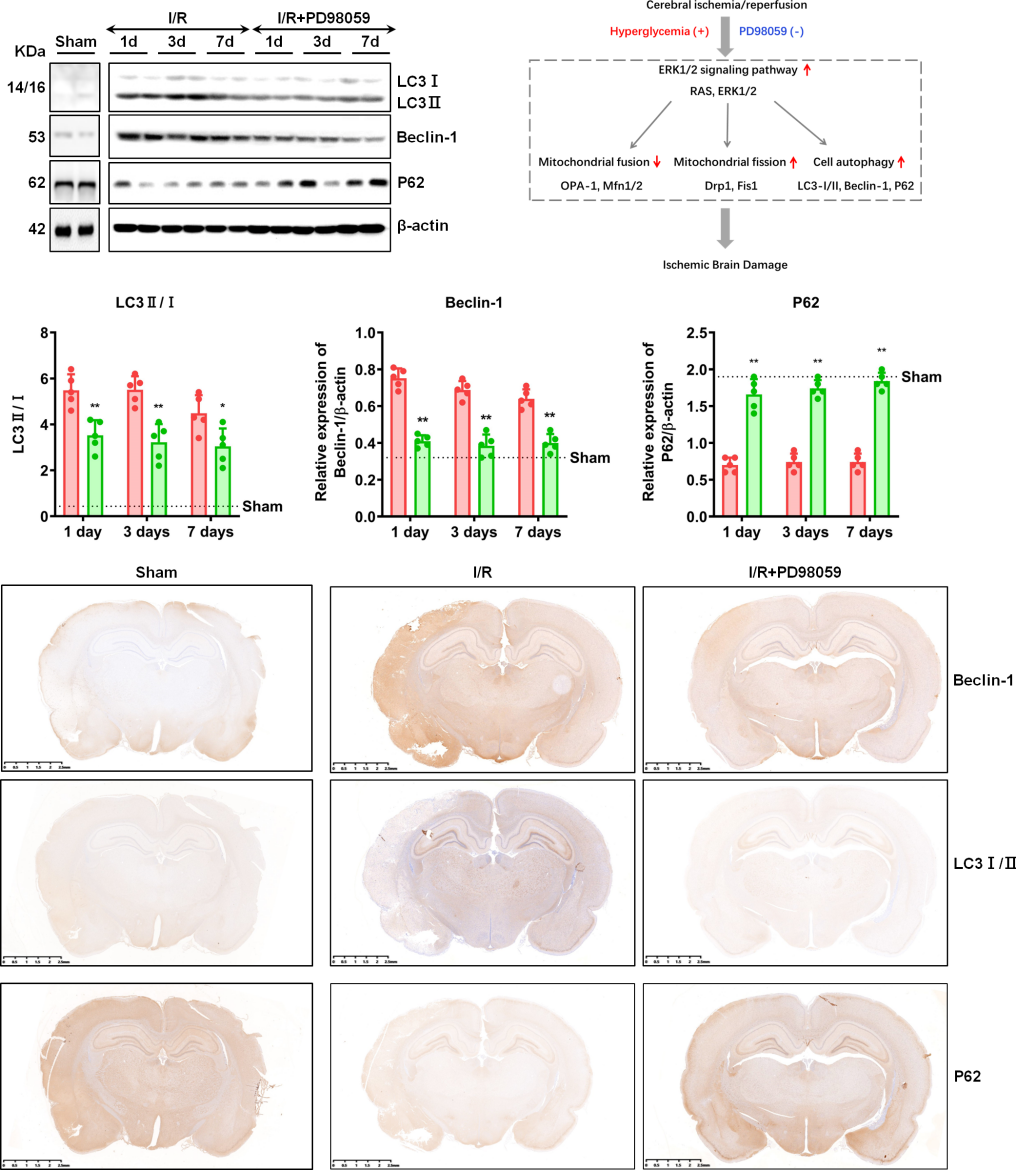
ERK1/2 inhibitor regulated cell autophagy. After I/R injury and administration of PD98059 in sham and diabetic rats, the expression of Beclin-1, LC3 I/II and P62 on the 1^st^, 3^rd^,and 7^th^ day were detected using western blot **(A)**, and gray values were determined by ImageJ **(B)**. The expression of Beclin-1, LC3 I/II and P62 were also verified by IHC **(C)**. Illustration for the role of hyperglycemia in aggravating cerebral I/R-induced brain injury *via* ERK1/2 signaling pathway **(D)**. In each group, n = 5, all the results are Mean ± SD, compared with I/R *p < 0.05, **p < 0.01.

Besides regulating mitochondrial fission, ERK1/2 pathway was also reported to be involved in cell autophagy (14, 23). To explore the effect of PD98059 on cell autophagy of cerebral I/R Rats, the expression of autophagy-related genes, Beclin-1, LC3 I/II and P62 were tested. The western-blot results showed that the decreased expression of Beclin-1 and LC3 I/II, and increased expression of P62 on the 1^st^, 3^rd^, and 7^th^ day in the I/R group + PD98059 than the I/R group (**Figures 5A, B**), which was also verified by IHC (**Figure 5C**), indicating that ERK1/2 inhibitor PD98059 protected brain tissue from cell autophagy (**Figure 5D**). Taken together, hypercemia aggravated celebral I/R associated brain injury *via* cell autophagy as well as mitochondrial fission.

## Discussion

Considerable studies have shown that cerebrovascular disease is the second most important clinical disease after cardiovascular disease with high morbidity and mortality (4, 5, 32). The World Stroke Organization (WSO) report shows that in 2019, over 120 million people die from stroke and related diseases each year, leading to a significant burden on society and the economy (32–35). The treatment and prevention of cerebrovascular disease is an urgent clinical issue (33, 35). Among the risk factors, diabetes mellitus (DM) plays a crucial role in cardiovascular disease (3, 4, 33).

Animal studies have shown that the higher the blood glucose, the worse the brain tissue damage (6, 7). Further increased blood glucose will cause death in all animals (13, 27), and it has been clinically confirmed that hyperglycemia aggravates ischemic tissue damage in the brain (30). In diabetic patients with cerebral ischemic stroke, the cerebral infarction area increased with decreased mortality and disability rates (4, 5, 9–11). However, the molecular mechanism of diabetes aggravating brain tissue damage is not clear.

Mitogen-active protease (MAPK) plays an important role in regulating cell growth, division, and death (14, 29). The MAPK family consists of three major components, c-Jun amino-terminal kinase (JNK), p-38, and extracellular signal-regulated kinase (ERK). ERK consists of ERK1 (44 kDa) and ERK2 (42 kDa), referred to as ERK1/2. The role of JNK and p-38 in cerebral ischemia injury has been largely clarified, and both of them lead to cell death after activation (13, 36–39). However, the role of ERK1/2 in cerebral ischemia injury has not been determined. On the one hand, ischemic preconditioning (one of the protective measures) resulted in a increase in p-ERK1/2 (phosphorylated ERK1/2) to protect nerve cells from damage (40, 41). On the other hand, the inhibition of the ERK1/2 pathway reduced the infarct area caused by middle cerebral artery infarction (12).

Our previous studies have found that the elevated blood glucose increased ischemic brain damage *via* activated mTOR and ERK1/2 signaling pathways in rats. Meanwhile, the treatment with mTOR inhibitor Rapamycin ameliorated the diabetes-enhanced brain injury through the suppressed phosphorylation ERK1/2 (17, 30, 31). In present study, more experiments were performed to investigate the mechanism of hyperglycemia related cerebral ischemia injury.

The ERK1/2 inhibitor PD98059 was a frequently-used drug to suppress ERK1/2 signaling pathway in animal models (42, 43). In this study, the neurological behavior scores and celebral I/R induced infarct volume treated with or without PD98059 were compared in DM rats. The suppression of ERK1/2 pathway could reduce nerve injury and alleviate the symptoms. Interestingly, the treatment with PD98059 reduced the severity of cerebral I/R- mediated brain damage, but shortened the recovery period. More important, compared with the Sham group, the mitochondrial membrane potential decreased in either the I/R group or the I/R + PD98059 group. The JC-1 ratio gradually recovered within 7 days, but the I/R + PD98059 group recovered faster than the I/R group, which was consistent with the cell apoptosis and neurobehavioral scores. In fact, even untreated with inhibitors, the rats recovered on the 7^th^ day. However, cellular apoptosis and mitochondrial fission are not reversible. These results indicated that the ERK1/2 pathway might play an indispensable role in celebral I/R brain damage in diabetic rats.

Two independent studies have shown that the RAS-ERK1/2 pathway phosphorylated Drp-1 leads to mitochondrial lysis and dysfunction, while the inhibition of ERK1/2 inactivates Drp-1 and promotes mitochondrial fusion (27, 28). Under normal conditions, mitochondria works in a dynamic balance between fusion regulated by Mfn1/2 and OPA1 and fission regulated by Drp-1 and Fis1 (22, 28, 29). Dysfunction of mitochondrial dynamic balance can lead to cell dysfunction, and inhibition of Drp-1 can protect nerve cells from damage (24, 25). Therefore, we hypothesized that diabetes or hyperglycemia aggravated the cerebral ischemia-activated ERK1/2 pathway. The active ERK1/2 kinases cause mitochondrial division, cleavage, and loss of function by promoting Drp-1 phosphorylation, leading to nerve cell death. In this experiment, the RAS expression and the phosphorylation of ERK1/2 and Drp-1 as well as Fis1 were increased in celebral I/R diabetic rats. However, the administration of ERK1/2 inhibitor PD98059 inhibited the expression of p-ERK1/2, p-Drp-1, and Fis1, but rescued the expression of mitochondrial fusion-related Mfn1/2 and OPA1, and mitochondrial DNA copy number and ATP content. In a word, hyperglycemia could result in neurological mitochondrial fission *via* modulation of ERK1/2 signaling pathay.

Accumulating evidence have shown that active ERK1/2 can also regulate cell autophagy during nutrient starvation (6, 14, 25, 26). Since diabetes aggravates brain tissue damage through the ERK1/2 pathway (31), we speculated that autophagy was probably one of the important consequences of hyperglycemic cerebral ischemia injury. According to our results, the expression of autophagy-related proteins, Beclin-1, LC3 I/II and P62 were overexpressed in celebral I/R group than Sham group. However, the use of PD98059 inhibited the expression of both Beclin-1, LC3 I/II and P62. In other words, diabetic cerebral I/R can also lead to increased cell autophagy by ERK1/2 signaling pathway.

## Conclusion

Collectively, we concluded that diabetic hyperglycemia might aggravate cerebral I/R-related brain damage *via* the activation of the ERK1/2 pathway, leading to cellular autophagy and mitochondrial fission, which eventually leads to the death of nerve cells.

## Data availability statement

The original contributions presented in the study are included in the article/supplementary material. Further inquiries can be directed to the corresponding author.

## Ethics statement

The animal study was reviewed and approved by the Animal Care and Use Committee of General Hospital of Ningxia Medical University.

## Author contributions

PL and XY, These two authors contributed equally to this work. Corresponding author: CH. All authors contributed to the article and approved the submitted version.

## Funding

This study is supported by National Natural Science Foundation of China (No.81660206 and No.82060156) to PL. National Natural Science Foundation of China (No.81860250) to XY. National Natural Science Foundation of China (No.82060237) to CH.

## Conflict of interest

The authors declare that the research was conducted in the absence of any commercial or financial relationships that could be construed as a potential conflict of interest.

## Publisher’s note

All claims expressed in this article are solely those of the authors and do not necessarily represent those of their affiliated organizations, or those of the publisher, the editors and the reviewers. Any product that may be evaluated in this article, or claim that may be made by its manufacturer, is not guaranteed or endorsed by the publisher.
